# Docosahexaenoic acid inhibits 12-*O*-tetradecanoylphorbol-13- acetate-induced fascin-1-dependent breast cancer cell migration by suppressing the PKCδ- and Wnt-1/β-catenin-mediated pathways

**DOI:** 10.18632/oncotarget.7301

**Published:** 2016-02-10

**Authors:** Chong-Kuei Lii, Jer-Wei Chang, Jia-Jing Chen, Haw-Wen Chen, Kai-Li Liu, Shu-Lan Yeh, Tsu-Shing Wang, Shu-Hui Liu, Chia-Han Tsai, Chien-Chun Li

**Affiliations:** ^1^ Department of Nutrition, China Medical University, Taichung, Taiwan; ^2^ Department of Health and Nutrition Biotechnology, Asia University, Taichung, Taiwan; ^3^ Institute of Molecular and Genomic Medicine, National Health Research Institute, Zhunan, Miaoli, Taiwan; ^4^ School of Nutrition, Chung Shan Medical University, Taichung, Taiwan; ^5^ Department of Nutrition, Chung Shan Medical University Hospital, Taichung, Taiwan; ^6^ Department of Biomedical Sciences, Chung Shan Medical University, Taichung, Taiwan

**Keywords:** fascin-1, TPA, docosahexaenoic acid, PKCδ, Wnt-1

## Abstract

Fascin-1, an actin-bundling protein, plays an important role in cancer cell migration and invasion; however, the underlying mechanism remains unclear. On the basis of a 12-*O*-tetradecanoylphorbol 13-acetate (TPA)-induced cell migration model, it was shown that TPA increased fascin-1 mRNA and protein expression and fascin-1-dependent cell migration. TPA dose- and time-dependently increased PKCδ and STAT3α activation and GSK3β phosphorylation; up-regulated Wnt-1, β-catenin, and STAT3α expression; and increased the nuclear translocation of β-catenin and STAT3α. Rottlerin, a PKCδ inhibitor, abrogated the increases in STAT3α activation and β-catenin and fascin-1 expression. WP1066, a STAT3 inhibitor, suppressed TPA-induced STAT3α DNA binding activity and β-catenin expression. Knockdown of β-catenin attenuated TPA-induced fascin-1 and STAT3α expression as well as cell migration. In addition to MCF-7, migration of Hs578T breast cancer cells was inhibited by silencing fascin-1, β-catenin, and STAT3α expression as well. TPA also induced Wnt-1 expression and secretion, and blocking Wnt-1 signaling abrogated β-catenin induction. DHA pretreatment attenuated TPA-induced cell migration, PKCδ and STAT3α activation, GSK3β phosphorylation, and Wnt-1, β-catenin, STAT3α, and fascin-1 expression. Our results demonstrated that TPA-induced migration is likely associated with the PKCδ and Wnt-1 pathways, which lead to STAT3α activation, GSK3β inactivation, and β-catenin increase and up-regulation of fascin-1 expression. Moreover, the anti-metastatic potential of DHA is partly attributed to its suppression of TPA-activated PKCδ and Wnt-1 signaling.

## INTRODUCTION

Metastasis is the most common cause of poor prognosis and worse survival rates in cancer patients. A meta-analysis reported that fascin, an actin-bundling protein, is consistently associated with increased risk of metastasis and mortality in numerous carcinomas, such as the gastric, colorectal, and breast cancers [1]. In mammals, the fascin protein family consists of three subtypes: fascin-1, fascin-2, and fascin-3 [2]. Fascin-1, is expressed in various cell types in normal tissues. Overexpression of fascin-1 significantly increases colon cancer cell motility and metastasis [3], whereas knockdown of fascin-1 results in decreased metastasis in a mouse xenograft model of prostate cancer [4] as well as in oral squamous cancer cells [5]. Fascin expression is induced by a variety of cytokines such as interleukin-6 (IL-6) and oncostatin M via transactivation of signal transducers and activators of transcription 3 (STAT3) in breast cancer cells [6]. In addition, transcription factors such as nuclear factor κB (NFκB) and hypoxia-inducible factor1 promote fascin gene transcription [7, 8].

12-*O*-Tetradecanoylphorbol 13-acetate (TPA), a mitogen and cancer promoter, induction of tumorigenesis is associated with increases in cell proliferation, epithelial to mesenchymal transition, and metastasis [9, 10]. Previous study revealed that the phosphorylation and distribution of fascin in actin filaments in filopodia and lamellipodia of the snail Helisoma are increased by TPA [11]. Furthermore, modulation of PKC activity by TPA leads to altered fascin localization and the formation of fascin-mediated microspikes in LLC-PK1 kidney epithelial cells and C2C12 skeletal myoblasts [12]. To date, however, whether the cancer promotion potency of TPA is related to changes in cellular fascin expression has not been fully elucidated.

The canonical Wnt/β-catenin signaling pathway is critical for steroidogenesis [13], proliferation and differentiation of tissue stem cells [14], and stabilization of brain endothelial tight junctions [15]. Upon Wnt binding with frizzled (FZ) and low-density lipoprotein-related protein (LRP), glycogen synthase kinase-3beta (GSK3β) is inactivated by phosphorylation at Ser9, which results in disruption of the Axin/APC/GSK3β/β-catenin protein complex. Subsequently, β-catenin is disassociated from the complex, which leads to the cytosolic accumulation of β-catenin [16, 17]. Thereafter, β-catenin translocates into the nucleus and binds with the T-cell factor (TCF) and the lymphoid enhancer-binding protein (LEF) family transcription factors, which promotes the transcription of target genes including cyclin D1 and fascin-1 [3, 18].

Disordered regulation of Wnt signaling is reported to be associated with several diseases, especially cancer. Wnt-1 overexpression promotes tumor progression in non-small-cell lung cancer [19], and hepatocellular carcinoma [20]. Wnt-1 is detected predominantly in invasive breast carcinomas [21] and the expression of Wnt-1 is required for epithelial-mesenchymal transition of breast cancer cells [22]. The first evidence for the cross talk between PKC and canonical Wnt signaling was provided by Goode et al. [23], who showed that activation of PKCα, β1, and γl results in phosphorylation of GSK3β at serine residues and consequent inactivation. Treatment with the PKC inhibitor calphostin C and dominant-negative PKC in 293 and C57MG cells diminishes Wnt-induced cytoplasmic accumulation of β-catenin and TCF/LEF-dependent transcriptional activation [24].

Docosahexaenoic acid (DHA), a n-3 polyunsaturated fatty acid, is well documented to have numerous health benefits in anti-angiogenesis [25], anti-thrombosis [26], and anti-tumorigenesis [27]. An *in vivo* study showed that fish oil supplementation decreases lung metastasis of breast cancer, and this protection may be through suppression of β-catenin expression and transactivation by DHA [28]. Recently, we reported that TPA-induced MCF-7 cell migration and invasion is suppressed by DHA at least in part via suppression of NFκB-induced MMP-9 expression [9]. However, whether fascin-1 plays a critical role in the anti-tumorigenic property of DHA remains unclear.

In this study, we intended to examine the underlying mechanism by which TPA increases fascin-1-driven cell migration in MCF-7 breast cancer cells. Moreover, we were interested to know whether changes in fascin-1 expression play a critical role in the inhibition of cancer cell migration by DHA.

## RESULTS

### Fascin-1 knockdown and DHA reduce TPA-induced MCF-7 cell migration

As measured by the 3-(4, 5-dimethylthiazol-2-yl)-2, 5-diphenyltetrazolium bromide (MTT) assay, the cell viabilities of MCF-7 cells treated with 100 ng/ml TPA alone and TPA plus 25, 50, and 100 μM DHA were 116.4% ± 1.8%, 113.9% ± 3.5%, 113.1% ± 1.6%, and 112.5% ± 13.9%, respectively, compared with the unstimulated controls (100%). These results indicated that there were no adverse effects on the growth of cells up to a concentration of 100 μM DHA in the presence of 100 ng/ml of TPA. In the following experiments, therefore, 100 ng/ml of TPA was used to induce the expression of fascin-1 and the highest concentration of DHA was set at 100 μM.

**Figure 1:**
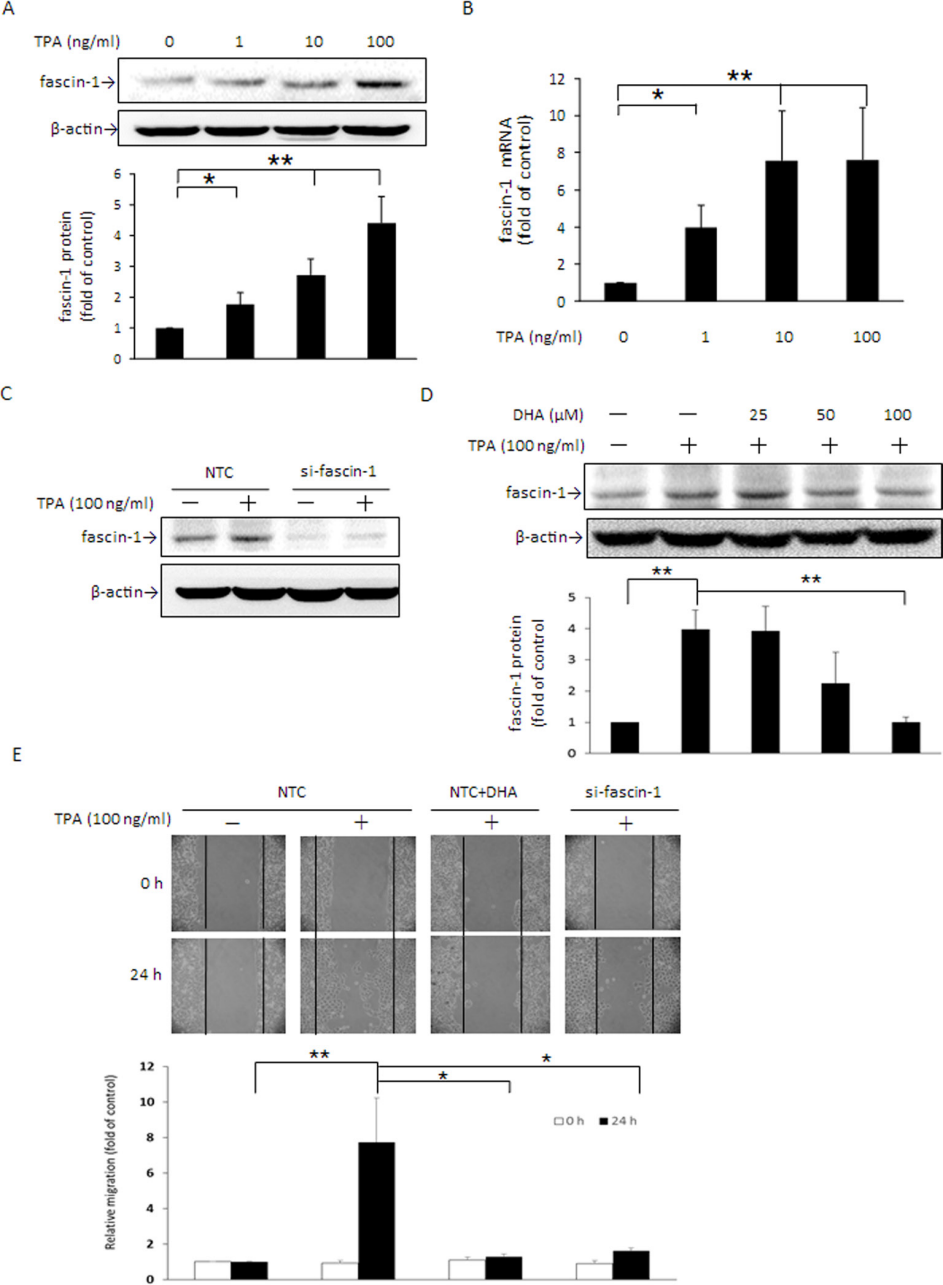
TPA induces fascin-1 expression in MCF-7 cells and fascin-1 siRNA abolishes TPA-induced cell migration MCF-7 cells were treated with various concentrations of TPA for 24 h. Fascin-1 protein (**A**) and mRNA (**B**) levels were determined. (**C**) Fascin-1 siRNA was used to silence fascin-1 mRNA in MCF-7 cells. After knockdown of fascin-1, the cells were treated with 100 ng/ml TPA for an additional 24 h. (**D**) Cells were pretreated with 0, 25, 50, or 100 μM DHA for 24 h followed by incubation with 100 ng/ml TPA for another 24 h. (**E**) After knockdown of fascin-1, the cells were transferred to the IBIDI culture insert and were then treated with or without 100 μM DHA for 24 h before being challenged with 100 ng/ml of TPA for an additional 24 h. Migration was observed by using a phase-contrast microscope at 100× magnification. One representative experiment out of three independent experiments is shown. Values are mean ± SD, *n* = 3. *p < 0.05 and **p < 0.01.

Fascin-1 has been recognized as an indicator of migration of colorectal and gastric cancer cells [1], and its high expression had strong association with basal-like phenotype and triple negative breast cancer (TNBC) patients [29]. To verify that fascin-1 plays an important role in breast cancer cell migration, MCF-7 cells were treated with TPA and Western blotting and the wound healing assay were performed. As shown, fascin-1 protein (Figure 1A) and mRNA (Figure 1B) expression were dose-dependently induced by TPA. After knockdown of fascin-1 expression by siRNA transfection, TPA-induced fascin-1 expression (Figure 1C) and MCF-7 cell migration (Figure 1E) were abrogated. When cells were pretreated with DHA, the TPA-induced increase in fascin-1 expression was dose-dependently attenuated (Figure 1D) and cell migration was suppressed as well (Figure 1E). These findings indicated that induction of fascin-1 is important in TPA-induced MCF-7 cell migration and that the anti-migration effect of DHA is likely associated with the suppression of this actin filament bundling protein.

### TPA up-regulates β-catenin and STAT3α expression and β-catenin siRNA abolishes TPA-induced STAT3α and fascin-1 gene expression in MCF-7 cells

**Figure 2:**
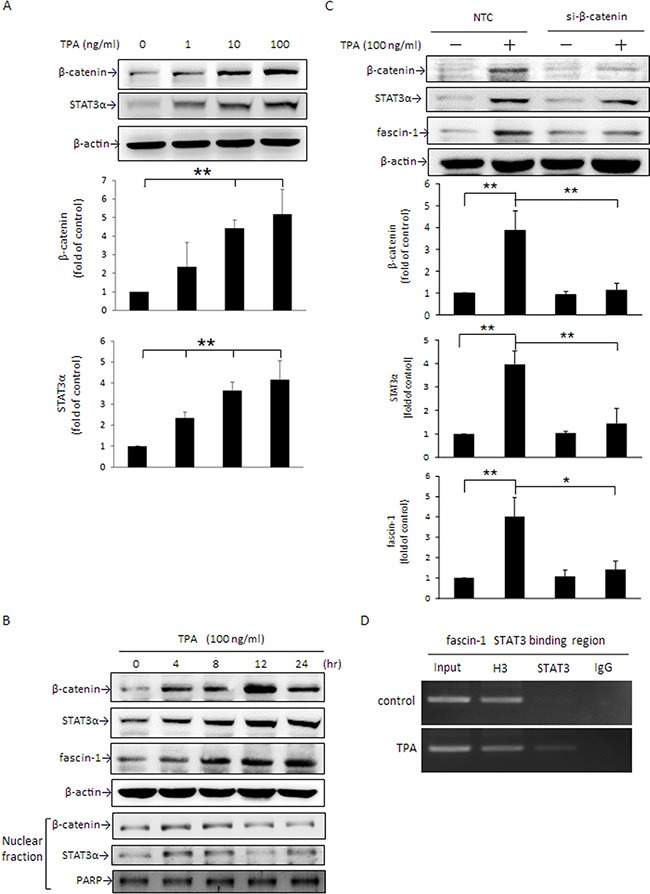
TPA induces cellular β-catenin and STAT3α protein expression and nuclear translocation and β-catenin siRNA abolishes TPA-induced STAT3α and fascin-1 expression in MCF-7 cells After treatment with various concentrations of TPA for 24 h (**A**) or with 100 ng/ml of TPA for 0–24 h (**B**), cellular β-catenin, STAT3α, and fascin-1 expression as well as nuclear (N) β-catenin and STAT3α expression were determined by Western blotting. (**C**) Cells were transfected with β-catenin siRNA or nontargeting control (NTC) and were then treated with 100 ng/ml of TPA for an additional 24 h. β-catenin, STAT3α, and fascin-1 proteins were determined. (**D**) MCF-7 cells were treated with 100 ng/ml of TPA for 6 h, and cell lysate was prepared for ChIP-PCR assay for STAT3 binding in fascin-1 gene in MCF-7 cells. “Input”, total input DNA; “H3”, DNA-protein complex pulled down by acetyl Histone H3; “STAT3”, DNA-protein complex pulled down by anti-STAT3; and “IgG”, DNA-protein complex pulled down by rabbit IgG antibody. H3 served as a positive control for STAT3 binding. Values are mean ± SD, *n* = 3. *p < 0.05 and **p < 0.01.

STAT3α acts as a key transcription factor in the modulation of fascin-1 gene expression in U87MG human glioblastoma cells [30]. β-Catenin overexpression dramatically induces STAT3α expression in human esophageal squamous carcinoma cells [31]. We thus next determined whether β-catenin-driven STAT3α expression participates in the TPA-induced fascin-1 expression in MCF-7 cells. As shown, cellular β-catenin and STAT3α levels were significantly increased by TPA in a dose- and time-dependent manner (Figure 2A and 2B). The induction of β-catenin and STAT3α appeared at 4 h and the increase in fascin-1 was first noted at 8 h after TPA treatment (Figure 2B). Consistent with these changes, nuclear β-catenin and STAT3α increased as well (Figure 2B). To further confirm that TPA-induced fascin-1 expression is mediated by the β-catenin/STAT3α pathway, cells were transiently transfected with β-catenin siRNA. As shown, TPA-induced STAT3α and fascin-1 expression (Figure 2C) and cell migration (Supplementary 1) were attenuated by silencing β-catenin expression. In addition, it was shown that STAT3 binding to the fascin-1 gene promoter was increased after treatment with TPA as demonstrated by ChIP assay (Figure 2D). These results suggest that β-catenin acts as an upstream component in STAT3α-increased fascin-1 transcription in response to TPA.

### Activation of STAT3α up-regulates β-catenin protein expression

**Figure 3:**
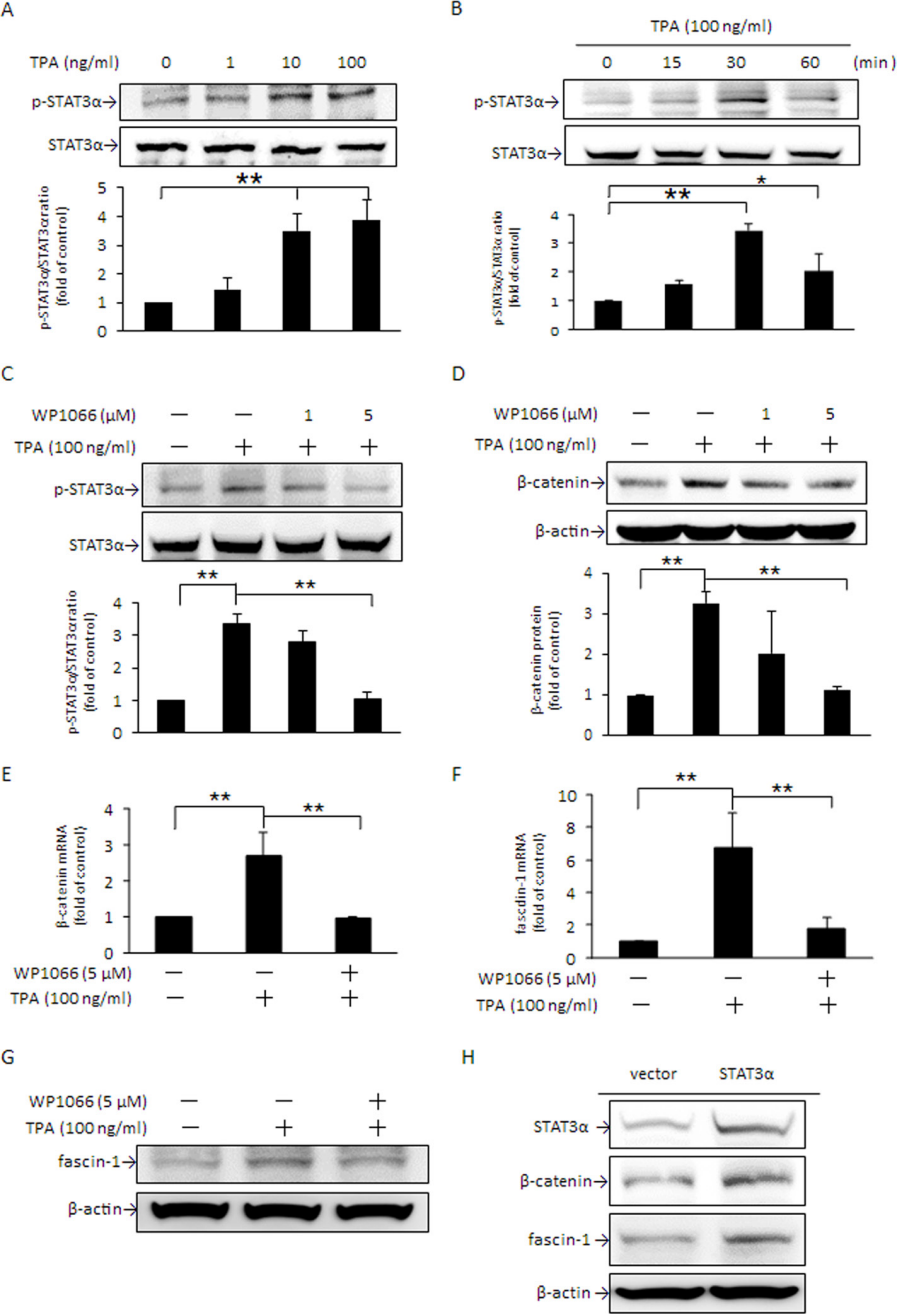
TPA increase in STAT3α phosphorylation up-regulates β-catenin and fascin-1 expression MCF-7 cells were treated with various concentrations of TPA for 30 min (**A**), were treated with 100 ng/ml of TPA for different time periods (**B**), or were pretreated with 1 or 5 μM WP1066 for 4 h followed by incubation with 100 ng/ml of TPA for another 30 min (**C**). STAT3α phosphorylation at Tyr705 was measured. Cells were pretreated with 1 or 5 μM WP1066 for 4 h followed by incubation with 100 ng/ml of TPA for another 24 h. The amounts of cellular β-catenin protein (**D**) were determined. Changes in β-catenin (**E**) and fascin-1 mRNA (**F**) and protein (**G**) were measured in cells pretreated with or without 5 μM WP1066 for 4 h, followed by incubation with TPA for another 18 h and 24 h, respectively. (**H**) Cells were transfected with STAT3 expression vector or pcDNA3.1 (**−**) control vector and were then treated with 100 ng/ml of TPA for an additional 24 h. STAT3α, β-catenin, and fascin-1 proteins were determined. One representative experiment out of three independent experiments is shown. Values are presented as mean ± SD, *n* = 3. *p < 0.05 and **p < 0.01.

In addition to STAT3 expression being induced by β-catenin, evidence indicates that β-catenin expression is mediated by STAT3 as well [32]. Two STAT3 binding sites have been identified on the β-catenin promoter [33]. In the presence of TPA, STAT3α activation through phosphorylation at Tyr705 was dose-dependently increased (Figure 3A) and the maximum increase was achieved at 30 min (Figure 3B). Pretreatment with 5 μM WP1066, a potent STAT3 inhibitor, attenuated TPA-induced phosphorylation of STAT3α (Figure 3C) and β-catenin protein (Figure 3D) and mRNA (Figure 3E) expression as well as cell migration (Supplementary 1). Accompanying these changes, the increase in fascin-1 mRNA and protein by TPA was significantly suppressed (Figure 3F and 3G). Moreover, overexpression of STAT3α by transient transfection with pcDNA3.1 (**−**)-STAT3α plasmid DNA apparently increased β-catenin and fascin-1 protein expression (Figure 3H). These findings supported that STAT3α activation is responsible for the TPA-induced increase in β-catenin expression. Taken together, the results noted in Figures 2 and 3 clearly indicated that, in the presence of TPA, STAT3α and β-catenin expression are mediated reciprocally.

### PKCδ mediates TPA-induced STAT3α activation and β-catenin expression in MCF-7 cells

**Figure 4:**
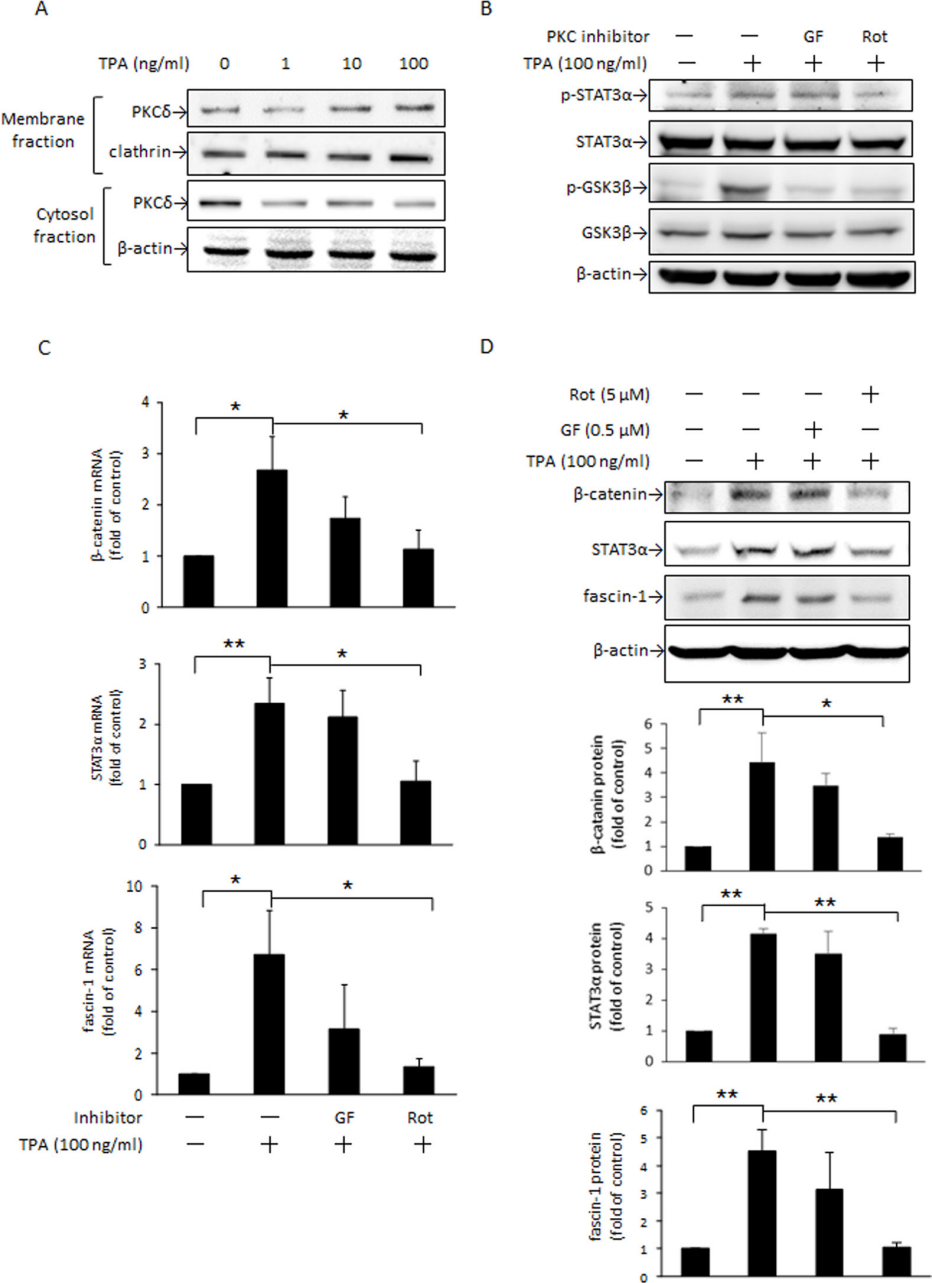
PKC is an upstream mediator of STAT3α and GSK3β phosphorylation induced by TPA MCF-7 cells were treated with various concentrations of TPA for 30 min and PKCδ in the plasma membrane and cytosolic fractions were determined (**A**). Cells were pretreated with or without 0.5 μM nonselective PKC inhibitor GF109203X (GF) or 5 μM PKCδ-specific inhibitor rottlerin (Rot) for 1 h followed by incubation with 100 ng/ml of TPA for another 30 min. STAT3α and GSK3β phosphorylation were measured (**B**). β-Catenin, STAT3α, and fascin-1 mRNA (**C**) and protein (**D**) levels were determined after 18 h and 24 h with TPA treatment, respectively. One representative experiment out of three independent experiments is shown. Mean ± SD, *n* = 3. *p < 0.05 and **p < 0.01.

PKC is a key kinase responsible for STAT3α activation [34]. Moreover, PKC inhibition of GSK3β activity by phosphorylating Ser9 leads to cytoplasmic accumulation of β-catenin in C57MG and 293 cells [24]. To verify whether PKC is involved in the TPA induction of β-catenin and fascin-1 protein expression in MCF-7 cells and which mechanisms are involved, we examined changes in PKC activation by TPA and STAT3α and GSK3β phosphorylation as well as β-catenin and fascin-1 expression in the presence of the nonselective PKC inhibitor GF109203X and the PKCδ-specific inhibitor rottlerin. As indicated, TPA dose-dependently increased PKCδ translocation from the cytosol to plasma membranes (Figure 4A). In the presence of rottlerin, but not GF109203X, the TPA-induced phosphorylation of STAT3α and GSK3β was inhibited (Figure 4B). The increases in β-catenin, STAT3α, and fascin-1 mRNA (Figure 4C) and protein (Figure 4D) expression, as well as cell migration (Supplementary 1) after TPA treatment were suppressed by rottlerin as well. These findings supported that PKCδ is an upstream mediator of STAT3α and GSK3β and plays a key role in the TPA induction of β-catenin and fascin-1 expression in MCF-7 cells.

### DHA suppresses TPA-induced PKCδ and STAT3α activation and GSK3β Ser9 phosphorylation as well as β-catenin and STAT3α expression

**Figure 5:**
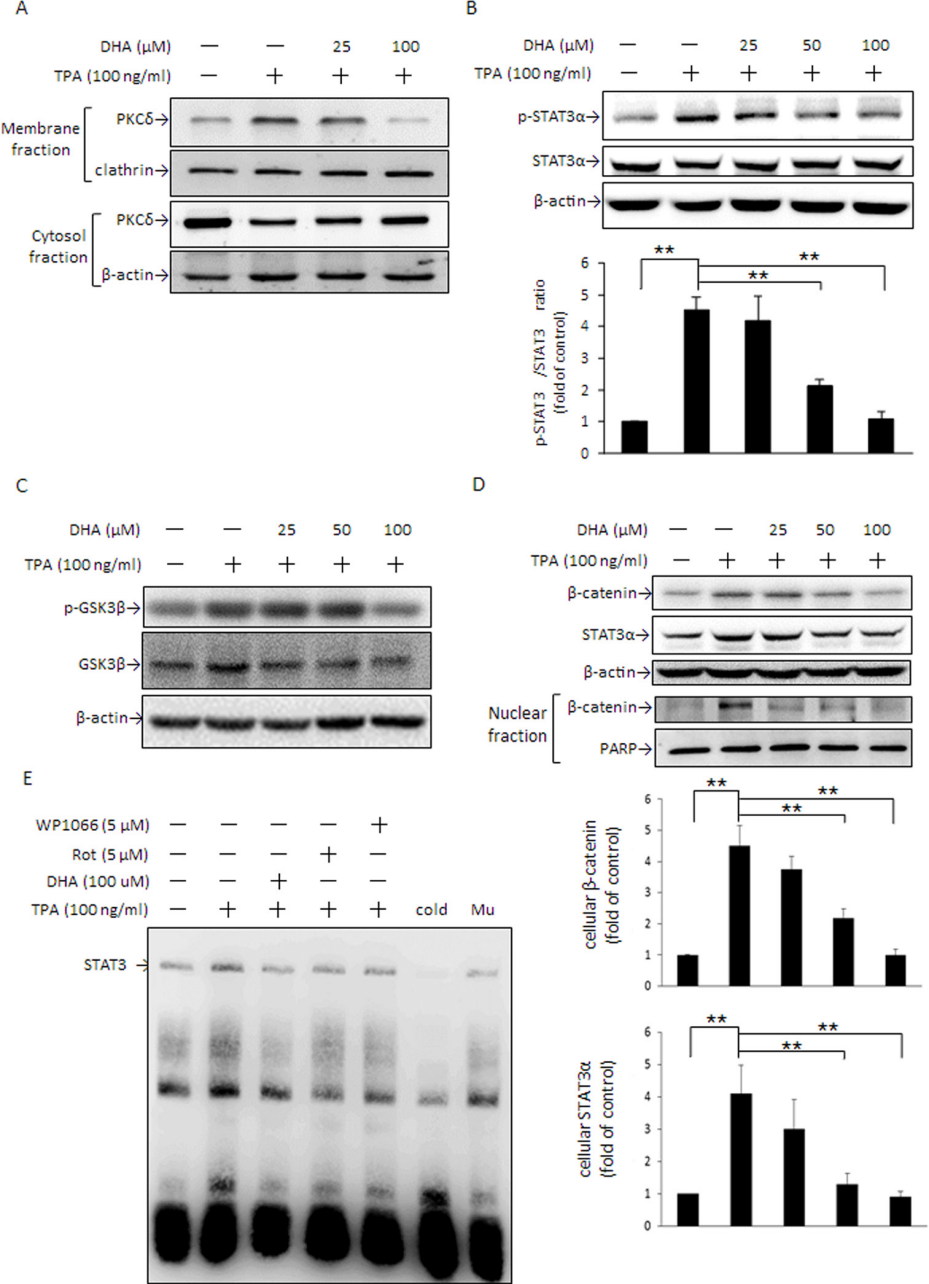
DHA inhibits TPA-induced PKCδ activation, STAT3 DNA binding activity, β-catenin, and STAT3α expression MCF-7 cells were pretreated with 0, 25, or 100 μM DHA for 24 h followed by incubation with 100 ng/ml of TPA for another 30 min. PKCδ in the plasma membrane and cytosol (**A**) and STAT3α (**B**) and GSK3β (**C**) phosphorylation were determined. (**D**) Total cellular β-catenin and STAT3α and nuclear β-catenin protein levels were determined in cells pretreated with various concentrations of DHA for 24 h followed by incubation with TPA for another 24 h and 4 h, respectively. (**E**) Cells were pretreated with 100 μM DHA for 24 h, 5 μM WP for 4 h, or 5 μM rottlerin (Rot) for 1 h, and then were incubated with TPA for another 6 h. Nuclear extracts (10 μg) were prepared for STAT3 nuclear protein DNA binding activity assay. To confirm the specificity of the nucleotide, 50-fold cold probe and mutant (Mu) were included in the EMSA. One representative experiment out of three independent experiments is shown. **p < 0.01.

As stated in Figure 1D and 1E, DHA inhibited TPA-induced fascin-1 expression and MCF-7 cell migration. Here, we further examined whether the inhibition of fascin-1-mediated migration by DHA was through the PKCδ-mediated STAT3α and GSK3β pathways. Upon DHA pretreatment, the TPA-induced PKCδ translocation from the cytosol to plasma membranes (Figure 5A) and the phosphorylation of STAT3α at Tyr 705 (Figure 5B) and of GSK3β at Ser9 (Figure 5C) were suppressed. Moreover, DHA dose-dependently attenuated TPA-induced β-catenin and STAT3α protein expression and β-catenin nuclear translocation (Figure 5D). The electrophoretic mobility shift assay (EMSA) revealed that DHA pretreatment abolished the TPA-induced STAT3 DNA-binding activity in MCF-7 cells (Figure 5E). A similar inhibition of STAT3α DNA-binding activity was observed in cells treated with rottlerin. These results clearly indicated that PKCδ is a key mediator of the DHA inhibition of TPA-induced β-catenin expression and STAT3α activity, which leads to suppressing fascin-1 expression.

### DHA and β-catenin, STAT3α, and fascin-1 knockdown suppress Hs578T cell migration

**Figure 6:**
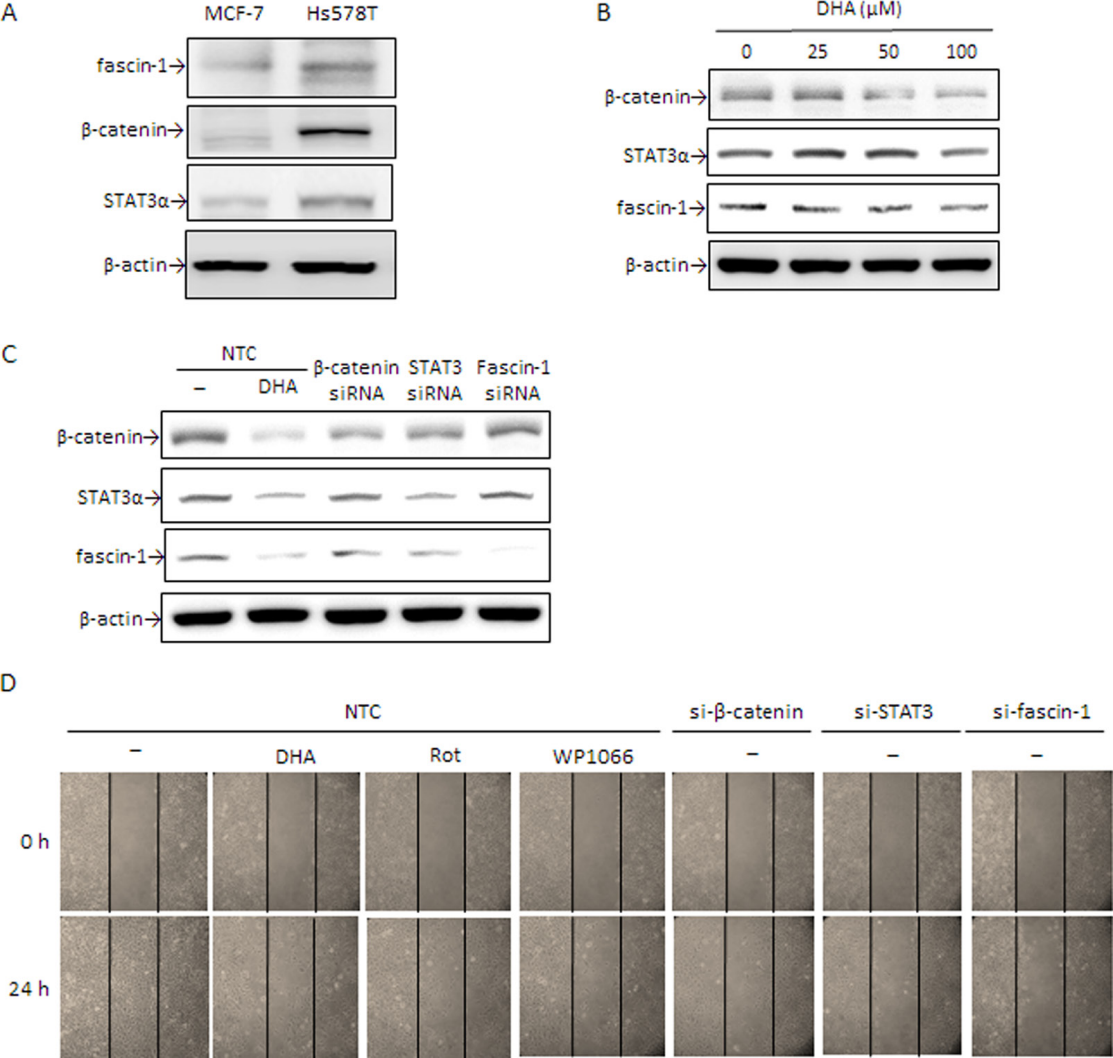
DHA and silencing of β-catenin, STAT3α, and fascin-1 suppress Hs578T cell migration (**A**) β-catenin, STAT3α, and fascin-1 expression in MCF-7 and Hs578T cells were determined by Western blotting. (**B**) Hs578T cells were treated with various concentrations of DHA for 24 h. (**C**) Cells were transiently transfected with β-catenin, STAT3, and fascin-1 siRNA or with nontargeting control (NTC) followed by treated with or without 100 μM DHA, 5 μM Rot or 5 μM WP1066 for an additional 24 h. Protein levels of β-catenin, STAT3α, and fascin-1 (C) as well as cell migration (**D**) were determined. One representative experiment out of three independent experiments is shown.

To further verify the importance of fascin-1 in cell migration, a malignant TNBC cell line Hs578T expressing high level of fascin-1, β-catenin, and STAT3α, is tested (Figure 6A). Expression of fascin-1, β-catenin, and STAT3α protein was dose-dependently attenuated by DHA (Figure 6B). Knockdown of fascin-1, β-catenin, and STAT3α e suppressed fascin-1 expression (Figure 6C). Concomitantly, β-catenin level was attenuated by silencing STAT3α. STAT3α expression, however, was slightly decreased by β-catenin silencing (Figure 6C). In consistent with a decrease in fascin-1 expression, knockdown of fascin-1, β-catenin, and STAT3α suppressed Hs578T cell migration (Figure 6D). Additionally, cell migration was inhibited by WP1066, and DHA (Figure 6D). These results further support that β-catenin and STAT3-mediated fascin-1 expression plays an important role in the migration of breast cancer cell and the anti-migration of DHA can be explained by its inhibition of fascin-1 expression.

### DHA attenuates TPA-induced Wnt-1 protein expression and extracellular secretion

**Figure 7:**
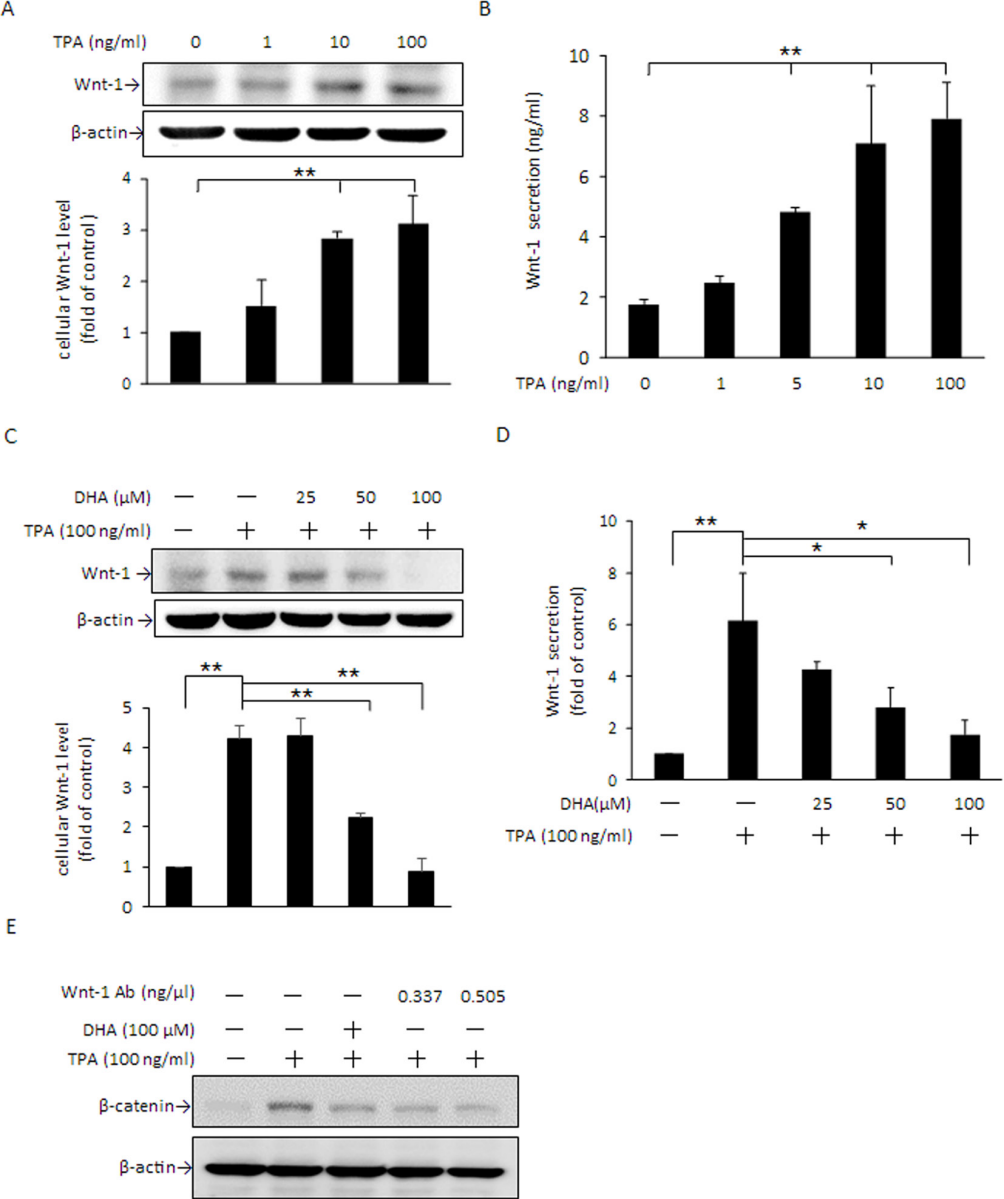
DHA inhibits TPA-induced Wnt-1 expression and extracellular secretion in MCF-7 cells (**A** and **B**) Cells were treated with 0–100 μM TPA for 24 h, and intracellular Wnt-1protein expression as well as extracellular secretion were determined by Western blotting and ELISA, respectively. (**C** and **D**) Cells were pretreated with 0, 25, 50, or 100 μM DHA for 24 h followed by incubation with 100 ng/ml TPA for an additional 24 h. The intracellular Wnt-1 protein levelandWnt-1 secretion into the media were determined. (**E**) Cells were pretreated with 0.337 or 0.505 ng/μl Wnt-1 antibody for 1 h or 100 μM DHA for 24 h followed by incubation with 100 ng/ml of TPA for another 24 h. The protein level of β-catenin was determined by Western blotting. Values are presented as mean ± SD, *n* = 3. *p < 0.05 and **p < 0.01.

In addition to PKCδ, the cellular β-catenin level can also be changed via Wnt-1 signaling, in which Wnt/FZ/LRP complex formation inactivates GSK3β activity and leads to an increase in β-catenin accumulation [16, 17]. Our results showed that 10 and 100 ng/ml TPA significantly induced Wnt-1 protein expression (Figure 7A) as well as extracellular secretion (Figure 7B). When cells were pretreated with DHA, the TPA-induced increase in Wnt-1 protein expression (Figure 7C) and Wnt-1 secretion (Figure 7D) were dose-dependently decreased. Moreover, TPA-induced β-catenin expression was attenuated by pretreatment with DHA or by blocking Wnt-1 signaling by Wnt-1 antibody (Figure 7E).

## DISCUSSION

Fascin-1is recognized as a crucial mediator in modulating embryogenesis [35], neurogenesis [36], and proliferation [37] and has been considered as a clinical prognostic marker of metastatic tumors [1]. Our previous study reported that TPA-induced MCF-7 breast cancer cell migration and invasion were suppressed by DHA [9]. However, it remained unknown whether fascin-1 was involved in the inhibition of cancer cell migration by DHA. Furthermore, the actual mechanism by which TPA up-regulates fascin-1-mediated cancer cell migration had not yet been fully elucidated. In this study, results revealed that fascin-1 is a determinant factor for breast cancer cell migration. In addition, we have reported for the first time that TPA induces MCF-7 cell migration through PKCδ and Wnt-1 signaling, which leads to activation of STAT3α, inactivation of GSK3β, and an increase in cellular β-catenin, and subsequently up-regulates fascin-1 gene transcription. Moreover, the anti-migration potency of DHA can be partly attributed to its effectiveness in suppressing TPA-induced activation of PKCδ and canonical Wnt-1 signaling.

STAT3α is a key transcription factor required for fascin-1 gene transactivation [30]. Constitutive activation of β-catenin in anaplastic lymphoma kinase (ALK)-positive anaplastic large cell lymphoma elevates STAT3 expression and activation [38]. In this study, a similar change in fascin-1 (Figure 1A) and β-catenin and STAT3α expression and nuclear translocation (Figure 2B, and 5D) in response to TPA was noted. When β-catenin was knocked down by siRNA, the TPA-induced cellular STAT3α and fascin-1 levels were attenuated (Figure 2C). Moreover, the increase in cell migration induced by TPA was suppressed by silencing fascin-1 (Figure 1E), β-catenin, and STAT3 expression (Supplementary 1). These results indicated that the TPA-induced fascin-1-dependent cell migration may be through the β-catenin-mediated STAT3α pathway. In addition to acting as an upstream mediator of STAT3, β-catenin expression and activation are increased by STAT3 overexpression as well (Figure 3H). Activation of STAT3 in colorectal cancer patients increases β-catenin transcription activity [39]. STAT3 silencing reduces β-catenin mRNA and protein levels in colorectal cancer cells [40]. A positive feedback loop, therefore, is believed to exist between β-catenin and STAT3 [41]. This explains why β-catenin siRNA inhibits TPA-induced STAT3α expression and why the STAT3α inhibitor WP1066 abrogates TPA-induced β-catenin expression as well (Figure 3D). Taken together, these results clearly indicate that this cross-interaction between STAT3α and β-catenin contributes to fascin-1-mediated MCF-7 cell migration induced by TPA. Knockdown STAT3α, β-catenin, and fascin-1 expression as well as treatment with WP1066 suppressed Hs578 cell migration (Figure 6D) further supporting the critical roles of STAT3α and β-catenin induced fascin-1 expression in breast cancer cell metastasis.

STAT3 activation is modulated by various mediators, of which PKC is a crucial one [34]. PKC acts as a crucial mediator in the modulation of numerous biological events in receptor desensitization, in regulating transcription, in mediating immune responses, and in regulating cell growth, as well as in modulating tumor development [42, 43]. To date, at least 11 PKC isozymes have been identified [44]. Among those, PKCδ is recognized to be important in gene transcription, cell cycle progression, and apoptotic cell death [45]. PKCδ is demonstrated to be responsible for STAT3 phosphorylation in an IL-6-dependent manner in both Hep3G human hepatocarcinoma cells and A431 epidermal squamous cancer cells [46]. In primary human monocytes, IL-13-induced 15-lipoxygenase expression is mediated by the PKCδ-dependent phosphorylation of STAT3 at Tyr705 and Ser727 [47]. A recent study showed that TPA-induced MMP-2 and MMP-9 activation is mainly via the PKCδ/ERK/NFκB pathways in MCF-7 cells [48]. Our previous study also reported that DHA down-regulates MMP-9 gene transcription and MCF-7 cell migration and invasion partly via the inhibition of the PKCδ/ERK1 pathway, which leads to inhibition of NFκB and AP-1 DNA binding activity [9]. In the present study, PKCδ translocation from the cytosol to plasma membranes was noted to be rapidly increased by TPA (Figure 4A). In the presence of the PKCδ inhibitor rottlerin, TPA-induced STAT3α phosphorylation at Tyr705 (Figure 4B), β-catenin, STAT3α and fascin-1 expression (Figure 4C and 4D), cell migration (Supplementary 1), and the DNA binding activity of STAT3α (Figure 5E) were attenuated. These findings indicated that PKCδ is an upstream mediator in the TPA-induced up-regulation of STAT3α-driven β-catenin and fascin-1 gene transcription. It needs to be addressed that, in addition to PKCδ, the possibility of other PKC isoforms played a role in GSK3β and STAT3α mediated fascin-1 expression cannot be excluded. A pseudosubstrate substrate peptide of PKCζ dose-dependently decreased TPA-induced phosphorylation of STAT3α and GSK3β in MCF-7 (Supplementary 2), suggesting that PKCζ may be important in TPA-induced cell migration. This report is the first to demonstrate that STAT3α is key in TPA-induced fascin-1-dependent cell migration.

In addition to the PKCδ/STAT3α pathway, the cellular β-catenin level is known to be associated with changes in GSK3β activity, which determines the formation of the axin/APC/GSK3β/β-catenin complex [16]. In addition to canonical Wnt-1 signaling, several kinases including PKC and p90RSK deactivate GSK3β by phosphorylating Ser9 of GSK3β, which leads to an increase in nuclear β-catenin translocation by disruption of the axin/APC/GSK3β/β-catenin complex [49, 50]. Activation of PKC leads to increased phosphorylation of GSK3β at Ser9, and β-catenin-mediated cyclin D1 expression is involved in scratching-induced injury and repair of bronchial epithelial cells [51]. In this study, changes in PKCδ activation (Figure 4A) by TPA were consistent with the increase in GSK3β inactivation by phosphorylation at Ser9 (Figure 4B) and nuclear β-catenin levels (Figure 2B). Moreover, GSK3β phosphorylation by TPA was suppressed by rottlerin (Figure 4B). These results indicate that, in addition to the PKCδ/STAT3α pathway, PKCδ-driven fascin-1 expression may also be through inactivation of GSK3β, which leads to β-catenin release from the axin/APC/GSK3β/β-catenin complex.

Autocrine Wnt signaling is known to play a vital role in numerous cellular events, including cell differentiation, cell migration, cell proliferation, and regeneration. Activation of Wnt signaling promotes MDA-MB-231, SKBR3, BT474, and MCF-7 breast cancer cell proliferation, whereas disruption of Wnt signaling decreases β-catenin-mediated proliferation and induces apoptosis [52]. Aberrant expression of Wnt-1 leads to activation of the Wnt-1/β-catenin signaling pathway, which is essential for MCF-7 breast cancer cell survival and metastasis [53]. Besides tumor development, enhanced Wnt/β-catenin signaling has been found to be positively correlated with the development of diabetes [54], rheumatoid arthritis [55], and Parkinson's disease [56]. In this study, we found that cellular Wnt- 1 expression and secretion (Figure 7A and 7B) in MCF- 7 cells were increased in response to TPA, and that the TPA-induced increase in β-catenin accumulation could be attenuated by blocking Wnt signaling with Wnt- 1 antibody (Figure 7E). These findings suggest that, in addition to the PKCδ/STAT3α and PKCδ/GSK3β pathways as stated above, Wnt-1 signaling also likely participates in the changes in cellular β-catenin in MCF-7 cells in response to TPA.

This raises the possibility that suppression of Wnt- and PKC-mediated signaling may be an effective approach in chemotherapy. Recently, several studies have reported that a number of phytochemicals, including curcumin, (**−**)-epigallocatechin-3-gallate (EGCG), and emodin, effectively suppress the proliferation and migration of Hep3B hepatocarcinoma cells and SW480 and SW620 colon cancer cells as well as the growth of xenograft tumors by inhibiting Wnt/β-catenin activation [57–59]. DHA is recognized to possess an anti-tumorigenic property by modulating the immune response, up-regulating antioxidant defense, inducing apoptosis, and inhibiting metastasis [60]. In this study, DHA was found to effectively inhibit TPA-induced Wnt-1 protein expression (Figure 7C) and extracellular secretion (Figure 7D). Moreover, the TPA-induced increase in GSK3β Ser9 phosphorylation (Figure 5C), cellular and nuclear β-catenin accumulation (Figure 5D) were attenuated by DHA. On the basis of these findings and the effective inhibition of DHA on fascin-1 expression (Figure 1D), it is reasonable to propose that DHA inhibition of fascin-1-mediated migration of MCF-7 cells is related to, at least in part, its interference with Wnt-1/β-catenin signaling.

PKC activity is known to be changed by a variety of dietary factors, including fatty acids [61], curcumin [62], and galangin [63]. For instance, DHA inhibits the translocation of PKCα and PKCε from the cytosol to the plasma membrane in PMA-treated NIH/3T3 cells [64]. Our recent work indicated that DHA inhibits PKCδ activation in TPA-treated MCF-7 cells; however, the actual working mechanism of the effect of PKCδ on the anti-metastasis action of DHA was not yet fully elucidated [9]. In this study, DHA decreases the membrane translocation of PKCδ from the cytosol under TPA treatment (Figure 5A). Accompanied by PKCδ transactivation, the TPA-induced increase in STAT3α and GSK3β phosphorylation (Figure 5B and 5C) as well as up-regulation of β-catenin (Figure 5D) and fascin-1 expression (Figure 1D) and cell migration (Figure 1E) in DHA-treated MCF-7 were attenuated. These findings suggest that PKCδ is likely to play a critical role in the down-regulation by DHA of fascin-1-dependent cell migration induced by TPA. Furthermore, it is likely that this down-regulation is through changes in the activation of GSK3β and STAT3α, which leads to suppression of cellular β-catenin and fascin-1 levels. Recently, the suppression of MDA-MB-231 breast cancer cell migration and invasion by grape seed extract was attributed to its down-regulation of β-catenin and fascin expression [65]. Furthermore, EGCG induces apoptosis and suppresses pancreatic cancer cell growth, metastasis by suppressing STAT3 transcription and activation [66]. In human THP-1 monocytic cells, the inhibition by anthocyanins of IFNγ-activated STAT3 decreases IL-6, tumor necrosis factor α (TNFα), and intercellular adhesion molecule 1 (ICAM-1) secretion [67]. Targeting to β-catenin and STAT3α is thus an effective approach against metastasis in human colon, prostate, and breast cancer cells [68, 69].

Fascin-1 expression is positively associated with the risk of several types of cancer incidence, including gastric, colorectal, esophageal, and breast carcinomas [1]. Decreases in the aberration of fascin-1 expression effectively inhibit the metastasis of cancer cells [5]. In addition to binding sites for STAT3 and TCF/LEF/β-catenin, binding sites for NFκB [7], hypoxia-inducible factor-1 [8], cAMP response element binding protein, and Smad [70] have been identified in the promoter of the fascin-1 gene. This raises the possibility that DHA acts to down-regulate fascin-1 expression by changing the transactivation of transcription factors other than STAT3 and TCF/LEF/β-catenin. Among those, NFκB and Smad are the two most attractive candidates because of the anti-inflammatory property of DHA, which is well documented to suppress the activation of these transcription factors. It has been reported that DHA inhibits TNFα- and TPA-induced ICAM-1 and MMP-9 expression in EA.hy926 and MCF-7 cells by suppressing IKK/NFκB signaling [9, 71]. DHA prevents hepatocellular injury in bile duct ligation causing cholestasis in rats through down-regulation of NFκB and TGFβ/Smad signaling [72]. Moreover, Yao and colleagues [7] recently demonstrated the existence of cross-talk between STAT3 and NFκB based on the findings that STAT3α siRNA abolishes NFκB binding to fascin-1 and subsequently inhibits metastasis of MKN45 human gastric carcinoma cells.

DHA is recognized as an extracellular molecule that manipulates intracellular physiological events via binding to a number of free fatty acid receptors (FFARs) [73]. It was shown that DHA exerts anti-proliferation and anti-inflammation through FFAR1 and FFAR4 [74, 75]. Because both FFAR1 and FFAR4 were identified to be located on the cell membrane of MCF-7 [76], it is interesting to explore whether these free fatty acid-specific receptors participate in DHA attenuation of TPA-induced PKCδ and Wnt-1 signaling pathways

**Figure 8:**
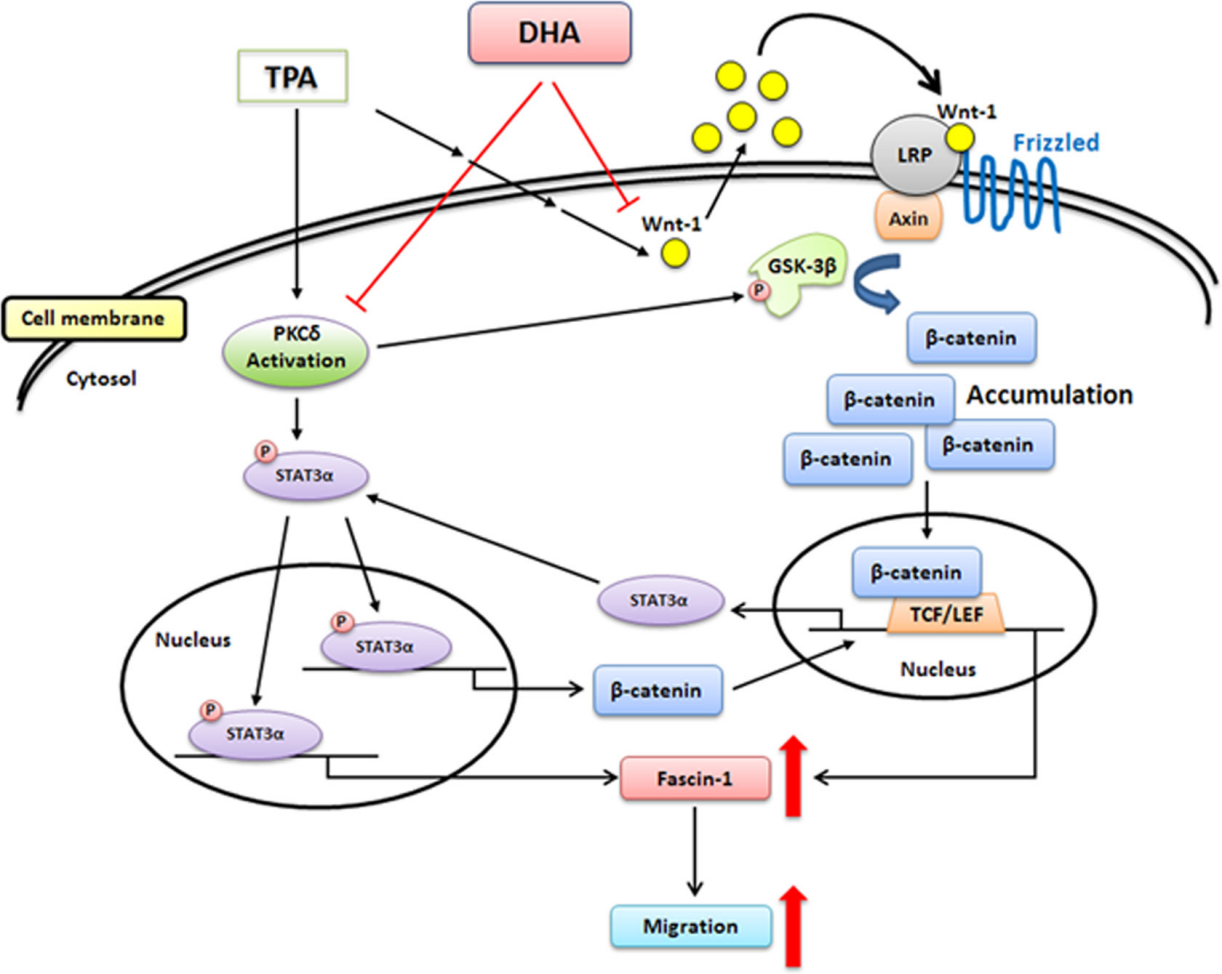
Scheme summarizing the TPA induction of fascin-1-dependent cell migration and the inhibition by DHA of TPA-induced MCF-7 cell migration via the down-regulation of PKCδ- and Wnt-1-mediated signaling pathways

In conclusion, TPA-induced migration of MCF-7 breast cancer cells is associated with the up-regulation of fascin-1 gene transcription, which is mediated through the activation of the PKCδ/STAT3α, PKCδ/GSK3β/β-catenin, and Wnt-1/β-catenin signaling pathways. DHA potently inhibits TPA-induced cell migration by attenuating PKCδ- and Wnt-1-mediated fascin-1 expression (Figure 8). Taken together, our findings provide new insights into the molecular mechanisms of the TPA induction of cell migration and the potency of DHA against metastasis of breast cancer cells.

## MATERIALS AND METHODS

### Reagents

DMEM, fetal bovine serum (FBS), penicillin–streptomycin solution, and 25% trypsin–EDTA were from GIBCO-BRL (GIBCO, Gaithersburg, MD); albumin, essentially fatty acid–free bovine serum albumin (BSA), sodium bicarbonate, calcium chloride, MTT, GF109203X, rottlerin and TPA were from Sigma-Aldrich (St. Louis, MO); STAT3 inhibitor III (WP1066) was from Merck (Darmstadt, Germany); DHA was from Cayman Chemical (Ann Arbor, MI); TRIzol reagent, Opti-MEM medium, and Lipofectamine RNAi MAX transfection reagent were from Invitrogen (Carlsbad, CA); antibodies against PKCδ (GTX61806; 78 KDa), Wnt-1 (GTX111182; 41 KDa), fascin-1 (GTX10051; 55 KDa), and β-actin (GTX109639; 42 KDa) were from GeneTex (Irvine, CA); antibodies against phospho-STAT3α (Tyr705)(#9138; 86 KDa), STAT3α (#9139; 86 KDa) and PARP (#9532; 116 KDa) were from Cell Signaling Technology (Danvers, MA); antibodies against β-catenin (#06–734; 92 KDa), GSK3β (#05–903; 47 KDa), phospho-GSK3β (Ser9)(#05–643; 47 KDa), STAT3 (#06–596), and EZ-Magna ChIP assay kit (#17–408) were from Millipore (Billerica, MA); antibody against clathrin (sc-6579; 192 KDa) was from Santa Cruz Biotechnology, Inc (Santa Cruz, CA) and the KAPA SYBR FAST qPCR Kit was from KapaBiosystems (Woburn, MA). The Wnt-1 ELISA kit was from USCN Life Science (Houston, TX).

### Cell culture and treatments

The human cell line MCF-7 and Hs578T were cultured and maintained in DMEM medium (pH 7.2) supplemented with 10% FBS, 1.5 g/l NaHCO_3_, 100 units/ml penicillin, and 100 μg/ml streptomycin at 37°C in a 5% CO_2_ humidified incubator. The culture medium was changed every other day. MCF-7 cells were grown to 80–90% confluence and were then treated with various concentrations of TPA (1–100 ng/ml) or were pretreated with 25–100 μM DHA for 24 h followed by incubation with TPA (100 ng/ml) in serum-free medium for the times indicated. The cell viability assay was performed as described in our previous study [77].

### Fatty acid preparation

DHA was freshly prepared and complexed with fatty acid–free BSA at a 6:1 molar ratio before addition to the culture medium. To prevent DHA-induced lipid peroxidation, 0.1% butylated hydroxytoluene and 20 μM α-tocopheryl succinate were added to the culture medium.

### Western blotting

Cells were washed twice with cold PBS and were harvested in 200 μl of 20 mM potassium phosphate buffer (pH 7.0). Cell homogenates were centrifuged at 9000 × *g* for 30 min at 4°C. The protein content of the supernatant was measured by using the Coomassie Plus Protein Assay Reagent kit (Pierce, Rockford, IL). Equal amounts of cellular proteins were electrophoresed in a sodium dodecyl sulfate (SDS)–polyacrylamide gel, and proteins were then transferred to polyvinylidene fluoride membranes (Millipore, Bedford, MA). The nonspecific binding sites in the membranes were blocked with 5% nonfat dry milk in 15 mM Tris–150 mM NaCl buffer (pH 7.4) at room temperature for 1 h. After blocking, the membranes were incubated with antibodies against fascin-1 (1:1000), β-catenin (1:1000), Wnt-1 (1:2000), PKCδ (1:1000), clathrin (1:1000), STAT3α (1:1000), phospho-STAT3α (1:1000), GSK3β (1:2000), phospho-GSK3β (1:1000), PPAR (1:1000) and β-actin (1:4000) at 4°C overnight. Thereafter, the membranes were incubated with the secondary peroxidase-conjugated anti-rabbit (1:6000) or anti-mouse IgG (1:5000) at 37°C for 1 h, and the immune-reactive bands were developed by use of the Western Lightning Plus-ECL Kit (PerkinElmer, Waltham, MA).

### Preparation of the membrane fraction for determining PKC translocation

The plasma membranes were prepared as described in our previous study [9]. After treatment, the cells were washed twice with cold PBS, after which cold buffer A (20 mM Tris, 30 μM Na_3_VO_4_, 2 mM MgCl_2_·6H_2_O, 2 mM EDTA, 0.5 mM EGTA, 2 mM PMSF, 1 mM DTT, 250 mM sucrose, and 10 μg/ml leupeptin) was added and the cells were scraped and lysed by use of a homogenizer (EyelaNazelax, Tokyo, Japan) on ice. The lysate was centrifuged at 100,000 × *g* for 1 h at 4°C. The supernatant was collected and used as the cytosolic fraction. The pellet was resuspended in cold buffer B (20 mM Tris, 30 μM Na_3_VO_4_, 5 mM MgCl_2_·6H_2_O, 2 mM EDTA, 0.5 mM EGTA, 2 mM PMSF, 1 mM DTT, 5 mM NaF, 10 μg/ml leupeptin, and 0.1% Triton X-100) and mixed by vortexing for 30 min. Samples were then centrifuged at 100,000 × *g* for 1 h at 4°C, and the supernatant was used as the membrane detergent-soluble fraction. PKC proteins in the two fractions were analyzed by Western blotting.

### RNA isolation and real-time PCR

Total RNA was isolated from MCF-7 cells by using TRIzol reagent (Invitrogen, Carlsbad, CA) according to the manufacturer's protocol. Amounts of 1 μg of total RNA were used to synthesize complementary DNA by use of SuperScript III Reverse Transcriptase (Invitrogen, Carlsbad, CA), and reverse transcription reactions were performed as described in our previous study [73]. Real-time PCR was performed on an ABI PRISM 7000 Sequence Detection System using the KAPA SYBR FAST qPCR Kit. Oligonucleotide primers for real-time PCR analysis were as follows: fascin-1 (forward, 5′-ATGGTCAAGTGCTGGATG-3′; reverse, 5′-GTAGAA GTTGGAGTCTGTAGG-3′), β-catenin (forward, 5′-ACAA GCCACAAGATTACAAG-3′; reverse, 5′-ATCAGCAG TCTCATTCCAA-3′), STAT3α (forward, 5′-AAGGAC ATCAGCGGTAAG-3′; reverse, 5′-AGATAGACCAGTGG AGACA-3′), and β-actin (forward, 5′-CGGCATCGTCACC AACTG-3′; reverse, 5′-TCTCAAACATGATCTGGGTC ATCT-3′). The condition of real-time PCR analysis was performed according to our previous study [9].

### Chromatin immunoprecipitation assay

The human fascin-1 (accession number: NM_003088.3) promoter sequence harbors STAT3 binding site located at −1070 to −1050 bp from the transcription start site, predicted by TFBIND website (http://tfbind.hgc.jp/). The STAT3 predicted binding sequences of fascin-1 promoter is consistent with that of a previous study [8]. TPA-untreated or -treated MCF- 7 cells were cross-linked with 1% formaldehyde, and cell lysates were sonicated to shear DNA to lengths between 200 and 800 bp by using Bioruptor^™^ system (Diagenode, Lie'ge, Belgium). ChIP was performed by use of the EZ-Magna ChIP assay kit (Millipore) according to the manufacturer's instructions. To briefly describe, antibodies against histone H3 (Millipore) and STAT3 (Millipore) were used to pull-down the sheared DNA. The DNA samples before immunoprecipitation were used as a template for input control. The PCR reactions used primers that flanked the STAT3 binding element sequence are as follows: upstream from **−**1124 to **−**1107 bp (5′-accttgtgggcagcctgt-3′) and downstream from **−**969 to **−**988 bp (5′-ATTCCCTGCAGACACCACCT-3′) of fascin-1 promoter. The expected PCR product size is 156 bp.

### Nuclear protein extraction and electrophoretic mobility shift assay

Cultures were pretreated with or without 100 μM DHA for 24 h, or 5 μM rottlerin for 1 h, or 5 μM WP1066 for 4 h before the addition of 100 ng/ml TPA for 6 h. After TPA treatment, MCF-7 cells were washed twice with cold PBS and were then scraped from the dishes with PBS. The preparation of nuclear protein and EMSA were performed as described [9]. Biotin-labeled double-stranded STAT3 consensus oligonucleotides (forward: 5′-TTGGCATGTGGGGAATGTCCAGGAAA-3′; reverse: 5′-TTTTCCTGGACATTCCCCACATGCCAA-3′) were designed according to the previous study [6] and used to measure STAT3 nuclear protein DNA-binding activity. Unlabeled double-stranded STAT3 oligonucleotide (5′-TTGGCATGTGGGGAATGTCCAGGAAA-3′) and mutant STAT3 oligonucleotide (5′-TTGGCATacttGGA ATGTCCAGGAAA-3′) were used to confirm the protein-binding specificity, respectively.

### RNA interference by small interfering RNA of fascin-1, STAT3 and β-catenin

Small interfering RNAs (siRNAs) for fascin-1, STAT3 and β-catenin were predicted and synthesized by MDbio Inc. (Taipei, Taiwan). MCF-7 cells were grown to 60–70% confluence in 35-mm plates and were transfected with fascin-1 and β-catenin siRNA or non-targeting siRNA (negative control) by use of Lipofectamine RNAi MAX Transfection Reagent (Invitrogen, Carlsbad, CA). Fascin-1 and β-catenin siRNA or negative siRNA were diluted in 50 μl Opti-MEM medium, respectively, and mixed with 2 μl of transfection reagent diluted in 98 μl of Opti-MEM medium. After incubation for 20 minutes at room temperature, the mixture was added to 800 μl of Opti-MEM medium and applied to the cells (1 ml/plate). After 8 h of transfection, the transfection reagent-containing medium was replaced with 10% FBS-containing DMEM medium for another 24 h, and then the cells were treated as indicated in the experimental design.

### Plasmid construction and transfection

The template clone of STAT3 (BC014482) was obtained from transOMIC technologies (Huntsville, AL, USA), and amplified the template using the following primer: forward, 5′-TGCTAGC GGACCCCTGATTTTAGCA-3′; reverse, 5′-GCTCGA GGGAACCACAAAGTTAGTAGTTT-3′. The PCR product was digested by NheI and XhoI restriction enzymes (NEB), and then the product was ligated into the same sites of pcDNA3.1 (**−**) expression vector. The MCF-7 cells were transfected with the pcDNA3.1-STAT3 plasmid and pcDNA3.1 control vector by using TransIT^®^-2020 transfection reagent, according to the manufacturer's instructions (Mirus Bio, Inc., Madison, WI, USA).

### Wound healing assay

For the MCF-7 cell migration assay, an IBIDI culture insert (IBIDI GmbH) was placed into a 35- mm culture dish and slightly pressed on top to ensure tight adhesion. An equal number of control and fascin-1-silenced MCF-7 cells (70 μl; 5 × 10^5^ cells/ml) were seeded into the two reservoirs of the same insert and incubated at 37°C in a 5% CO_2_ humidified incubator. After 24 h, the insert was gently removed, creating a gap of about 500 μm. Cells were then cultured in DMEM medium without FBS and incubated with 100 ng/ml TPA for another 24 h. Cells were then photographed (100×magnification) to monitor cell migration into the wounded area, and the width of the cell-free zone (distance between the edges of the injured monolayer) was calculated.

### Extracellular Wnt-1 secretion

MCF-7 cells were grown to 80–90% confluence and were then treated with various concentrations of TPA or pretreated with 25 to 100 μM DHA for 24 h followed by incubation with 100 ng/ml TPA for an additional 24 h. Afterward, 100-μl aliquots of culture medium were taken and analyzed for Wnt-1 by using the Wnt-1 ELISA kit according to the manufacturer's instructions (USCN Life Science Inc., Houston, TX).

### Inhibition of Wnt-1 signaling

MCF-7 cells were plated in 6-cm dishes and maintained in normal medium. When the cells had grown to approximately 80% confluence, they were pretreated with Wnt-1 antibody (0.337 and 0.505 ng/μl) for 1 h or were pretreated with 100 μM DHA for 24 h followed by incubation with 100 ng/ml TPA for an additional 24 h at 37°C. After 24 h of incubation, the total proteins were collected and analyzed by Western blotting.

### Statistical evaluation

Values are means ± standard deviation. Difference between group means was compared by Student's *t*-test. (version 10.0; SPSS, Chicago, IL). *P* values < 0.05 or < 0.01 were considered to be statistically significant. Experiments were repeated three times (*n* = 3).

## SUPPLEMENTARY MATERIALS FIGURES


