# Supplementary material for: Docosahexaenoic acid inhibits 12-O-tetradecanoylphorbol-13- acetate-induced fascin-1-dependent breast cancer cell migration by suppressing the PKCδ- and Wnt-1/β-catenin-mediated pathways
Source: Oncotarget. 2016 Feb 10;7(18):25162–79. doi: 10.18632/oncotarget.7301 (PMC5041895; doi:10.18632/oncotarget.7301)
Supplement: Supplementary file 1 [file oncotarget-07-25162-s001.pdf]

Docosahexaenoic acid inhibits 12-*O*-tetradecanoylphorbol-13-acetate-induced fascin-1-dependent breast cancer cell migration by suppressing the PKCδ- and Wnt-1/β-catenin-mediated pathways

Supplementary Materials

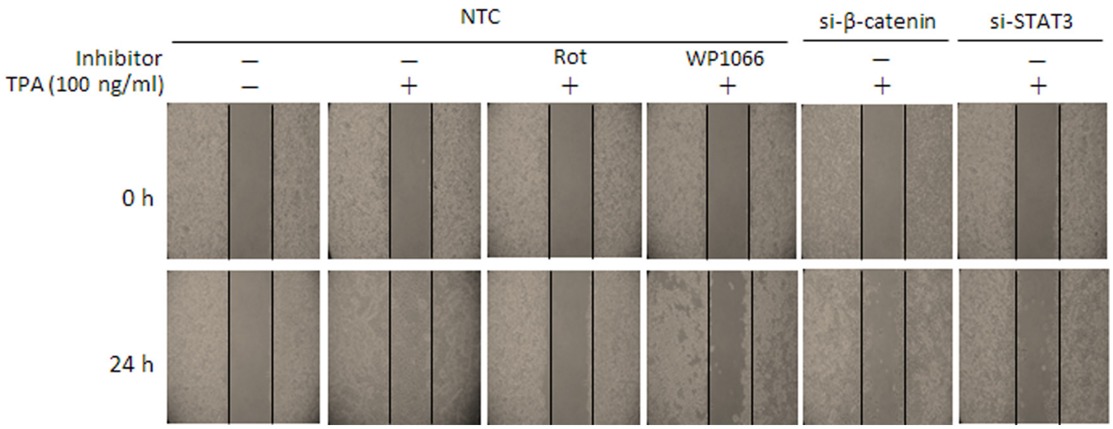

Supplementary Figure S1: Rottlerin (Rot), WP1066 (WP), β-catenin siRNA, and STAT3 siRNA suppressed TPA-induced MCF-7 cell migration. Cells were transfected with nontargeting control (NTC), β-catenin siRNA or STAT3 siRNA. After siRNA transfection, cells were incubated with 5 µM Rot for 1h or 5 µM WP1066 for 4 h followed by challenging with 100 ng/ml of TPA for an additional 24 h. Migration was observed by using a phase-contrast microscope at 100× magnification.

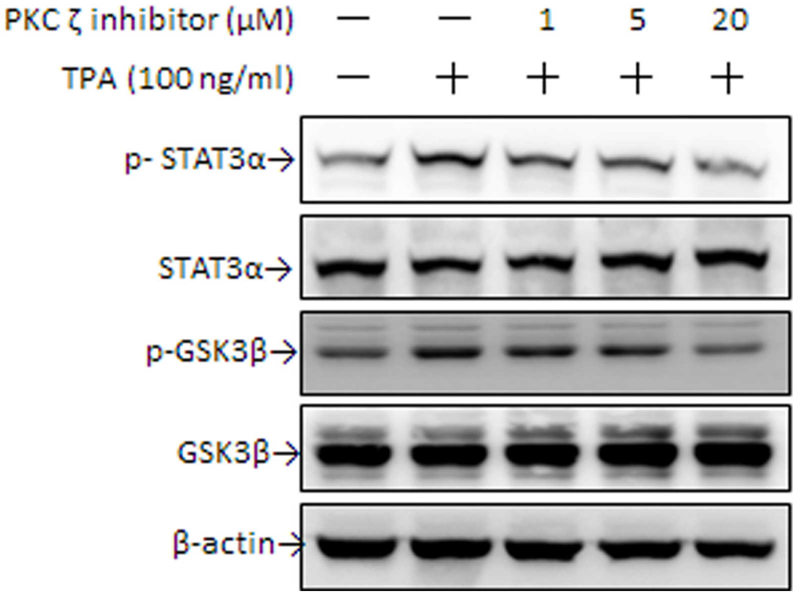

Supplementary Figure S2: PKC ζ inhibitor inhibited TPA-induced STAT3α and GSK3β phosphorylation in a dose-dependent manner in MCF-7 cells. Cells were pretreated with 0, 1, 5, or 20 µM PKC ζ inhibitor for 1 h followed by incubation with 100 ng/ml of TPA for another 30 min. STAT3α and GSK3β phosphorylation were measured.
